# Correction: “I did not know it was a medical condition”: Predictors, severity and help seeking behaviors of women with female sexual dysfunction in the Volta region of Ghana

**DOI:** 10.1371/journal.pone.0228696

**Published:** 2020-01-30

**Authors:** The PLOS ONE Staff

## Notice of Republication

This article was republished on January 17, 2020, to correct errors in the author list. The publisher apologizes for this error. Please download this article again to view the correct version. The originally published, uncorrected article and the republished, corrected articles are provided here for reference.

## Supporting information

S1 File Originally published, uncorrected article. (PDF)

S2 File Republished, corrected article. (PDF)
