# Supplementary material for: “I did not know it was a medical condition”: Predictors, severity and help seeking behaviors of women with female sexual dysfunction in the Volta region of Ghana
Source: PLoS One. 2020 Jan 13;15(1):e0226404. doi: 10.1371/journal.pone.0226404 (PMC6957185; doi:10.1371/journal.pone.0226404)
Supplement: Attachment — Submitted filename: Response to Reviewers_version 2.docx [file pone.0226404.s003.docx]

**Comment**

Reviewer #1: Dear Authors,
I have read your revised paper and it reads better than the previous one. I feel the contribution of this study will be better appreciated if it were grounded in a theory/model.

**Response**

We addressed this comment in the limitations for future studies in the discussion section:

“Future research endeavors should also consider qualitative research methodologies that will better contextualize and support the study findings, through the use of narratives and theoretical frameworks. The contribution of this paper may be better appreciated if it were supported with qualitative theories/frameworks/models.”

Minor issues:

**Comment**
• The sentence on sexual problems in response to Comment 1 in the introduction is long. You may want to reconsider for clarity.

**Response**

We have made some edits as follows (these are also tracked in the document):

Sexual problems are often multifactorial and can be classified into four broad categories, which are: biomedical (biological factors such as pregnancy, injury and disability, cardiovascular and other chronic diseases that can interfere with intercourse); intrapsychic (psychological elements within the individual, that influence their sexual expression e.g., values learned about one’s body, nudity, “where babies come from”, puberty, etc); interpersonal (factors that influence one’s ability to engage in sexual relationships, such as communication difficulties with a partner, the extent to which partners share compatible visions of sex, eroticism, pleasure etc.); and socio-cultural/economic/political (‘blueprints’ for sexual norms, beliefs, values, practices and attitudes that are created and imposed on an individual based on the ‘moral code’ of their society. Includes religion.) [4, 15]. These four factors often overlap, and rarely occur in isolation to cause the sexual dysfunction [4].

**Comment**
• Since the term human sexuality is introduced in the work for the first time, I am not quite sure about the appropriateness of the current location, i.e., discussion (pages 21-22). In my view, it will better fit for the introduction.

**Response**

We revised the discussion to be more specific to sexual function and reads as follows:

Though human sexual function has requisite biological underpinnings, it is usually experienced as a complex interaction of biological, intrapsychic, interpersonal and socio-cultural/economic/political factors [4, 15].

**Comment**
• A recap of the main objective(s) in the first paragraph of the conclusion would be useful.

**Response**

We updated the conclusion to read as follows:

The study’s main objective was to describe the prevalence and severity of female sexual dysfunction (FSD) amongst a group of Ghanaian women in the outpatient setting of the predominantly rural Volta region of Ghana. Additionally we determine the predictors of FSD severity and care seeking behaviors of women with the condition.We found that FSD is highly prevalent amongst women in rural settings of the Ghanaian community, with lubrication, pain and satisfaction disorders being the most associated with severity.

**Comment**

• It’s surprising the authors find the suggestion to proofread the paper for clarity a contradiction to other segments of the online reviewer form. Overall assessment of an article (in relation to the intelligibility and writing standard) is not coterminous with specific errors noted in same.

**Response**

We must have misinterpreted the following comment by the reviewer:

“There are language problems and sloppiness...”

Thank you for the clarification.

**Comment**
I want to commend the authors for the point-by-point responses to the specific issues raised in the article and I look forward to reading the published version. Thank you for your fine contribution.

All my very best!

**Response**

We appreciate your insights
